# Supplementary material for: Horizontal Cervix as a Novel Sign for Predicting Adhesions on the Posterior Extrauterine Wall in Cases of Placenta Previa
Source: J Clin Med. 2019 Dec 4;8(12):2141. doi: 10.3390/jcm8122141 (PMC6947443; doi:10.3390/jcm8122141)
Supplement: Supplementary file 1 [file jcm-08-02141-s001.pdf]

Supplemental Figure 1 Intraoperative image of posterior extrauterine wall adhesions.

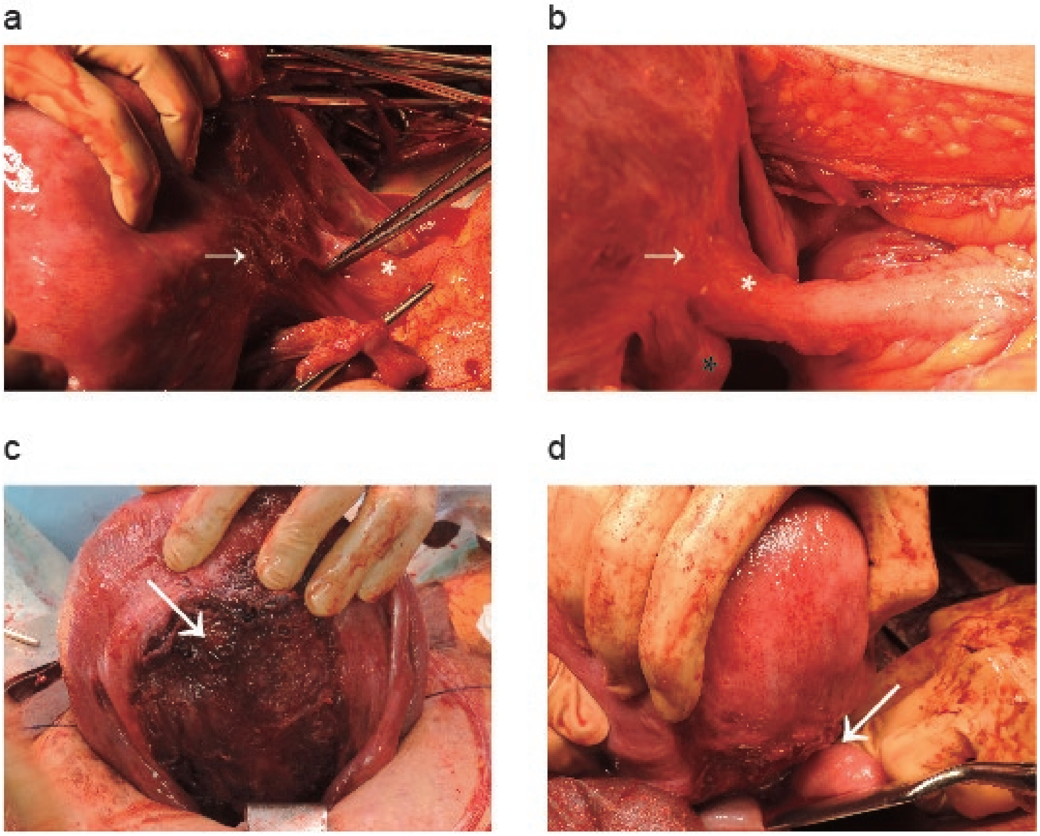

We showed four patients with intraoperative findings of posterior extrauterine wall adhesions. The cervical horizontal sign was positive in all patients. Although it was difficult to confirm by histopathological examination, we considered that these adhesions were caused by endometriosis.

(a) Intraoperative view of cesarean delivery at gestational week 34. Cervical canal angle was investigated at gestational week 30 and the angle was 0°. Intraoperative finding showed strong adhesion between the rectum (shown by the white asterisk) and the posterior extrauterine wall posterior wall (shown by the white arrow).

Intraoperative view of cesarean delivery at gestational week 35. Cervical canal angle was investigated at gestational week 28 and the angle was -10°. Strong adhesions among the rectum (shown by the white asterisk), the posterior extrauterine wall posterior wall (shown by the white arrow), and left ovary were observed (shown by the black asterisk).

(b) Intraoperative view of cesarean delivery at gestational week 37. Cervical canal angle was

investigated at gestational week 30 and the angle was -20°. Intraoperative finding revealed that extensive dissection of adhesions was performed for extracorporeal elevation due to the posterior extrauterine wall adhesion (shown by the white arrow).

(c) Intraoperative view of cesarean delivery at gestational week 37. Cervical canal angle was investigated at gestation week 27 and the angle was -15°. Intraoperative finding revealed that exteriorization of the uterus could not be performed due to the posterior extrauterine wall adhesion (shown by the white arrow).
